# Pushing health data access boundaries - adapting NHS governance for 3 challenges: clinical free-text, safe commercial access and AI development

**DOI:** 10.23889/ijpds.v9i5.1649

**Published:** 2024-09-18

**Authors:** Amy Tilbrook

**Affiliations:** 1 University of Edinburgh

This presentation will demonstrate expansions to TRE governance frameworks developed to facilitate safe data access in three advanced areas: commercial access, clinical free-text, and innovation with AI/Machine Learning.

NHS research governance frameworks - originally set up for clinical trials and expanded into the TRE and secondary data use space – are not an immediate fit for advanced techniques in data science research. Over 2 years, public, expert and user engagement approaches were utilised to support developing frameworks within DataLoch, a Health and Social Care TRE and data access service in South-East Scotland.

Surveys, workshops and continuous user and data controller feedback allowed the testing of tools and assessments to adapt traditional NHS governance structures. These frameworks utilise the Five Safes framework to make NHS data controllers supportive of innovative research in the public and health service’s benefit.

The presentation will summarise the challenges faced for one TRE and researchers working to support these areas, and the frameworks that are now are being tested within an NHS Scotland Health Board. The development and implementation demonstrates that governance structures can be adapted collaboratively and paves the way for more flexible TRE governance to allow the use of NHS patient data to remain legally and ethically robust while supporting advanced techniques of analysis.

